# Drug spectrum and disproportionality signals for diabetes insipidus in FAERS: multi-method analysis, JADER cross-database assessment, and PubMed-based case-level evidence

**DOI:** 10.3389/fphar.2026.1840741

**Published:** 2026-09-04

**Authors:** Yiqi Li, Yu Zhou, Jinghe Tian, Deyi Wei, Xuan Bi, Hengyi Liu, Lin Yang

**Affiliations:** Bishan Hospital of Chongqing Medical University, Bishan Hospital of Chongqing, Chongqing, China

**Keywords:** diabetes insipidus, disproportionality analysis, drugs, FAERS, JADER, pharmacovigilance, spontaneous reporting systems

## Abstract

**Objective:**

The aim of this study is to characterize reporting patterns and non-treatment drug safety signals for diabetes insipidus (DI) in the FDA Adverse Event Reporting System (FAERS), evaluate signal stability using crude, adjusted, and shrinkage-based disproportionality methods, and assess signal reproducibility in the Japanese Adverse Drug Event Report (JADER).

**Methods:**

We analyzed FAERS (2004Q1–2025Q4) and JADER (2004–2025) reports. DI cases were defined by MedDRA Preferred Terms “Diabetes insipidus” and “Nephrogenic diabetes insipidus”; central DI-related lower-level terms mapped to “Diabetes insipidus” and were not separately retrievable. Drugs used for DI diagnosis/treatment or arginine vasopressin (AVP)-deficiency states were excluded during preprocessing. All eligible non-treatment primary suspect drug–DI pairs were screened, and the main multi-method summary focused on the 50 most frequently reported drugs. Published cases were summarized as case-level evidence, not comparative risk meta-analysis.

**Results:**

FAERS contained 3,444 Preferred Term (PT)-defined DI reports; 3,325 entered the main non-treatment analysis. Raw annual counts increased, whereas normalized reporting proportions fluctuated. Complete screening identified 112 crude reporting odds ratio (ROR)-positive drug–DI pairs. Among the frequency-ranked top 50, 47 met crude ROR criteria, 38 remained positive after adjustment, 32 met empirical Bayes geometric mean (EBGM) criteria, and 40 met information component (IC) criteria; in addition, 36 were positive in at least three methods. In JADER, 696 DI reports entered cross-database assessment; eight drugs met crude ROR criteria, eight met adjusted ROR criteria, three met EBGM criteria, and three met IC criteria. Lithium, hydrocortisone, and propofol were positive across all four JADER methods.

**Conclusion:**

After treatment-drug exclusion, DI reports clustered mainly among nervous system, antineoplastic/immunomodulating, and systemic hormonal agents. Several non-treatment drugs showed persistent disproportionality, but JADER support was limited. These findings are hypothesis-generating signals, not causal evidence.

## 1 Introduction

Diabetes insipidus (DI) is a rare disorder of water balance characterized by hypotonic polyuria and polydipsia caused by impaired arginine vasopressin (AVP) secretion or action (Tomkins et al., 2022; Atila et al., 2022). It is commonly classified as central DI, resulting from insufficient AVP secretion from the hypothalamic–pituitary axis, or nephrogenic DI, resulting from renal resistance to AVP (Flynn et al., 2025; Levtchenko et al., 2025). Both forms can lead to dehydration, hypernatremia, and serious clinical complications when water access or fluid replacement is inadequate (Levtchenko et al., 2025). Central DI, now often framed as AVP deficiency, is usually treated with desmopressin, whereas nephrogenic DI, or AVP resistance, is managed by correcting the underlying cause, maintaining adequate water intake, reducing solute load, and using selected adjunctive therapies when appropriate (Levtchenko et al., 2025; Atila et al., 2024).

Drug-associated DI is uncommon but clinically important because it may be overlooked outside well-established drug–event pairs. Lithium-induced nephrogenic DI remains the prototypical example (Grünfeld and Rossier, 2009). Long-term lithium exposure can impair urinary concentrating capacity through aquaporin-2 downregulation and collecting-duct remodeling (Kortenoeven et al., 2009). Other reported or suspected associations are more heterogeneous. Dexmedetomidine and perioperative anesthetic or sedative agents have been linked to transient DI-like polyuria in case reports and reviews (Van Decar et al., 2022). Selected antipsychotic, antiepileptic, antineoplastic, and immunomodulating agents have also been reported in individual cases or pharmacovigilance analyses (Kumar et al., 2023; Esteves-Ferreira and Rosinha, 2023). These observations suggest that drug-associated DI may arise through different mechanisms, including central AVP suppression, renal AVP resistance, tubular injury, and immune-mediated hypothalamic–pituitary dysfunction.

Spontaneous reporting databases provide a useful platform for screening rare drug–event combinations that are difficult to evaluate in clinical trials. The FDA Adverse Event Reporting System (FAERS) is a large post-marketing pharmacovigilance database containing individual case safety reports submitted by healthcare professionals, consumers, and manufacturers, and it has been widely used to generate real-world drug safety signals (Khaleel et al., 2022; Hauben et al., 2021; Potter et al., 2025). However, disproportionality analyses based on spontaneous reports cannot establish causality or estimate incidence, and their interpretation is influenced by reporting behavior, drug utilization, coding practices, and clinical context. These limitations are particularly relevant for DI because drugs used to diagnose or treat DI can be co-reported with the event itself, and because central and nephrogenic DI are not always separable in public MedDRA Preferred Term (PT)-level data (Chen et al., 2021; Contreras-Salinas et al., 2024; Wang B. et al., 2025; Li et al., 2025).

A recent FAERS-based study on drug-induced DI by Lv et al. (2026) analyzed reports from 2004Q1 to 2024Q4 and identified 2,189 DI cases and 71 positive drug signals using reporting odds ratio (ROR) and BCPNN methods, with lithium and dexmedetomidine among the most prominent signals. This prior study provided an important database-wide overview. Several questions remained unresolved, including how to handle indication-related co-reporting from DI diagnostic or treatment drugs, whether signals persist after covariate adjustment and shrinkage-based stabilization, whether FAERS-derived signals show reproducibility in an independent national database, and how MedDRA coding affects interpretation of central versus nephrogenic DI.

The present study was designed to address these issues rather than to repeat FAERS screening alone. We used FAERS as the primary database and the Japanese Adverse Drug Event Report (JADER) as an independent cross-database assessment source. DI cases were defined using a prespecified MedDRA term audit, and drugs used for DI diagnosis or treatment or AVP-deficiency states were removed during initial drug preprocessing. We focused on non-treatment primary suspect drugs, evaluated signal stability using crude ROR, covariate-adjusted ROR, empirical Bayes geometric mean (EBGM), and information component (IC), and performed supplementary PS + SS, ROR-ranked, and clinical-context sensitivity analyses. Finally, published case-level evidence was integrated as structured clinical context rather than as comparative risk estimation. This design aimed to distinguish frequently reported non-treatment signals from those with stronger multi-method and cross-database support while keeping causal interpretation appropriately cautious.

## 2 Materials and methods

### 2.1 Study design

This retrospective observational pharmacovigilance study used individual case safety reports (ICSRs) to assess reporting associations between drugs and DI. FAERS was used as the primary database for signal screening, and the JADER database was used for cross-database assessment of signal reproducibility. Because both databases contain publicly available and de-identified reports, individual informed consent and institutional review board approval were not required.

The analysis had five components: extraction and deduplication of FAERS and JADER reports; definition of DI cases using predefined MedDRA PTs selected through a MedDRA term audit; drug-name standardization, drug-role restriction, and exclusion of drugs used for DI diagnosis or treatment; disproportionality analyses using crude ROR, covariate-adjusted ROR, EBGM, and information component (IC); and supplementary analyses, including normalized annual reporting proportions, primary plus secondary suspect drug sensitivity analysis, ROR-ranked signal prioritization, clinical-context sensitivity analysis, and PubMed-based case-level evidence mapping.

**FIGURE 1:**
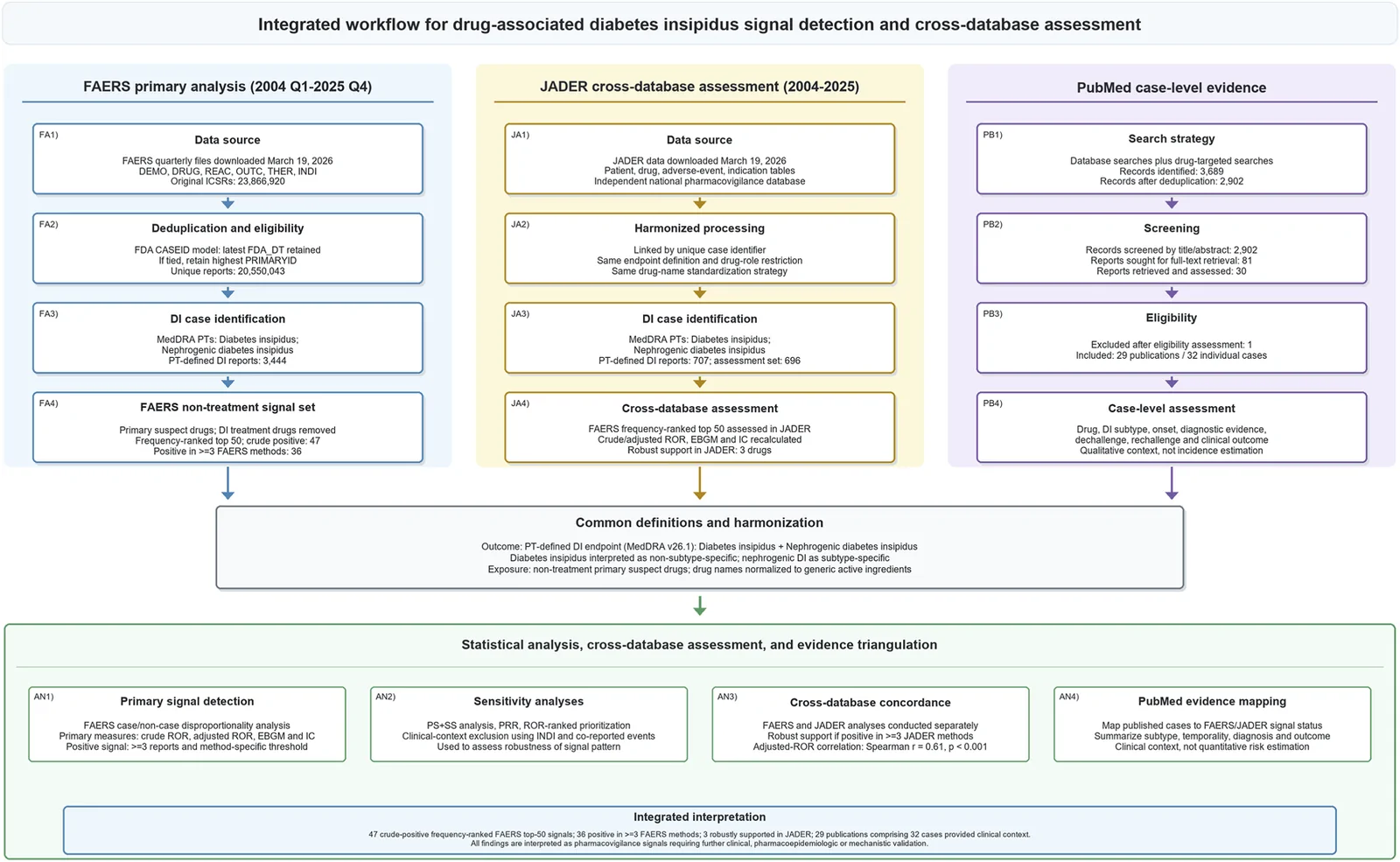
Study design and analytical framework. Integrated workflow for identifying and prioritizing drug-associated diabetes insipidus (DI) pharmacovigilance signals using FAERS, JADER, and published case-level evidence. FAERS reports from 2004Q1 to 2025Q4 were used for the primary analysis, and JADER reports from 2004 to 2025 were used for cross-database assessment. DI cases were defined using predefined MedDRA Preferred Terms based on a MedDRA version 26.1 term audit, including “Diabetes insipidus” and “Nephrogenic diabetes insipidus.” Drug exposure was restricted to non-treatment primary suspect drugs after exclusion of drugs used for DI diagnosis or treatment, and drug names were harmonized to generic active ingredients. The main FAERS signal set focused on the 50 non-treatment primary suspect drugs with the highest DI-related reporting frequencies and was evaluated using crude ROR, adjusted ROR, EBGM, and IC. Sensitivity analyses included PS + SS analysis, PRR analysis, ROR-ranked prioritization, clinical-context exclusion using indication and co-reported adverse-event fields, and PT-level subtype assessment. The same FAERS frequency-ranked top 50 drugs were assessed in JADER using the four disproportionality methods, with robust cross-database support defined by positivity in at least three JADER methods. Published case-level evidence was used to characterize DI subtype, temporality, diagnostic evidence, dechallenge, rechallenge, and clinical outcome. All findings were interpreted as hypothesis-generating pharmacovigilance signals rather than confirmed causal associations.

All analyses were designed for pharmacovigilance signal detection. They were not intended to estimate incidence, absolute risk, or causality. Published case reports were used only to provide qualitative clinical context for selected drug–DI signals (Figure 1).

### 2.2 Data sources and extraction

FAERS is a publicly available spontaneous reporting database maintained by the U.S. Food and Drug Administration for post-marketing drug safety surveillance. Quarterly FAERS files from 2004Q1 through 2025Q4 were downloaded from the FDA website on 19 March 2026. The DEMO, DRUG, REAC, OUTC, THER, and INDI tables were imported when available. FAERS adverse events were coded using MedDRA terminology, whereas drug names were reported as submitted and may include generic names, brand names, abbreviations, misspellings, salt forms, or other variants.

JADER is a spontaneous reporting database maintained by the Pharmaceuticals and Medical Devices Agency in Japan. JADER data from 2004 through 2025 were downloaded from the PMDA website on 19 March 2026. The patient, drug, adverse-event, and indication tables were imported. JADER adverse events were coded using MedDRA terminology, and drug names were analyzed using available standardized ingredient fields where possible.

For FAERS, tables were linked using PRIMARYID. Case-level deduplication followed FDA recommendations: when multiple records shared the same CASEID, the record with the most recent FDA_DT was retained; when FDA_DT was identical, the record with the highest PRIMARYID was retained. Reports without valid drug or adverse-event information were excluded from the corresponding drug-event analyses.

For JADER, tables were linked using the database-specific case identifier. The same endpoint definition, drug-role restriction, treatment-drug exclusion strategy, drug-name normalization approach, and signal criteria were applied to JADER where corresponding variables were available.

### 2.3 Study population and report classification

The study population consisted of deduplicated FAERS and JADER reports during the study period that contained at least one reported drug and at least one adverse-event term. No restrictions were applied by age, sex, country or region, reporter type, seriousness, or outcome unless specified for a given analysis.

Reports were classified as PT-defined DI cases if the adverse-event table contained at least one predefined MedDRA PT included in the primary DI endpoint. All other eligible reports were treated as non-cases and served as the reference group for case/non-case disproportionality analyses. The case definition was based only on adverse events recorded in the adverse-event table. DI terms recorded only as indications, medical history terms, or concomitant conditions were not used to define cases unless the same DI PT was also recorded as an adverse event.

### 2.4 Adverse-event definition and MedDRA term audit

The endpoint of interest was DI, defined using MedDRA PTs. A MedDRA version 26.1 term audit was performed before case extraction. The audit reviewed DI-related PTs and lower-level terms (LLTs), their PT mapping relationships, inclusion or exclusion status, observed availability in FAERS and JADER, and the rationale for endpoint selection. The full audit is provided in the Supplementary Material.

The primary PT-defined DI endpoint included “Diabetes insipidus” and “Nephrogenic diabetes insipidus.” These PTs were selected because both are PT-level adverse-event terms directly retrievable from public FAERS and JADER adverse-event tables. “Diabetes insipidus” captures non-subtype-specific DI coding, whereas “Nephrogenic diabetes insipidus” preserves the only consistently available subtype-specific acquired DI PT.

Central DI-related LLTs, including “central diabetes insipidus,” “pituitary diabetes insipidus,” “hypothalamic diabetes insipidus,” “antidiuretic hormone deficiency,” and “vasopressin deficiency,” were mapped to the broader PT “Diabetes insipidus” and are not available as separate PT-level endpoints in public FAERS or JADER data. Therefore, reports coded as “Diabetes insipidus” were interpreted as non-subtype-specific DI rather than definite central DI.

Congenital DI-related PTs, including “Congenital central diabetes insipidus” and “Congenital nephrogenic diabetes insipidus,” were reviewed during the MedDRA audit but were excluded from the primary endpoint because they represent congenital disorders rather than acquired or drug-associated DI.

### 2.5 Drug definition and drug-name standardization

Drug exposure was identified from the drug table. The primary analysis was restricted to drugs recorded as primary suspect (PS) to improve drug-attribution specificity. To assess the influence of drug-role restriction, a sensitivity analysis was performed after including both primary suspect and secondary suspect (SS) drugs.

Drugs used for DI diagnosis or treatment or AVP-deficiency states were removed during the initial drug preprocessing stage before signal calculation, top-50 selection, and cross-database assessment. Excluded terms included vasopressin, desmopressin, DDAVP, argipressin, pitressin, lypressin, and direct synonymous or brand-related terms captured during standardized drug-name processing. When a DI report contained multiple remaining suspected drugs, each eligible drug was analyzed as a separate drug–DI pair.

Drug names were standardized to generic active ingredients before analysis using MedEx or available standardized ingredient fields. Brand names, spelling variants, capitalization differences, and salt forms were mapped to active ingredients where possible. If a drug name could not be reliably standardized or interpreted as a valid drug entity, it was excluded from drug-specific signal analysis. Fixed-dose combination products were mapped to active ingredients when unambiguous; otherwise, they were retained as reported.

### 2.6 Descriptive analysis and missing-data handling

Descriptive analyses were performed for DI-related reports in FAERS and JADER. Variables included reporting year, age, sex, reporter type, country or region when available, seriousness, reported outcomes, and suspected drugs. Categorical variables were summarized as frequencies and percentages. Continuous variables were summarized using medians and interquartile ranges when appropriate.

Missing demographic or clinical values were not imputed. For descriptive summaries, percentages were calculated using the number of reports with available information for the corresponding variable as the denominator unless an “unknown” or “unspecified” category was explicitly coded and retained. The number of reports with missing or unavailable age, sex, reporter country, and outcome information was reported where relevant.

For temporal analyses, annual counts of PT-defined DI reports were normalized by the total number of deduplicated FAERS reports in the same reporting year and expressed as DI-related reports per 100,000 FAERS reports. This analysis was used to distinguish changes in absolute DI report counts from changes associated with overall FAERS reporting volume.

### 2.7 Disproportionality analysis

Disproportionality analysis was used to screen for disproportionate reporting patterns in FAERS and JADER (Barcelos et al., 2019; Beau-Lejdstrom et al., 2019). For each drug–DI pair, a two-by-two contingency table was constructed. The number of reports containing both the drug of interest and DI was defined as a; reports containing the drug of interest and non-DI events as b; reports containing DI without the drug of interest as c; and reports containing neither the drug of interest nor DI as d.

The crude ROR and 95% confidence interval (CI) were calculated as follows:

$$ROR=\frac{a/c}{b/d}=\frac{a\times d}{b\times c},$$

$$95\%CI=\exp\left[\ln\left(ROR\right)\pm 1.96\times \sqrt{\frac{1}{a}+\frac{1}{b}+\frac{1}{c}+\frac{1}{d}}\right].$$

A positive crude ROR signal was defined as at least three DI-related reports for the corresponding drug–DI pair and a lower bound of the 95% CI greater than 1. Drug–DI pairs with fewer than three DI reports were not interpreted as positive signals because of sparse-cell instability. ROR magnitude was interpreted as disproportionate reporting rather than causality or clinical risk.

Crude ROR screening was first conducted among all eligible non-treatment PS drug–DI pairs. All pairs meeting the predefined crude ROR signal criterion were exported as a complete supplementary signal table. For the main visualization and multi-method comparison, we focused on the 50 remaining PS drugs with the highest DI-related reporting frequencies after treatment-drug exclusion. This frequency-ranked top-50 strategy prioritized signals supported by larger report counts. As an additional prioritization analysis, we also evaluated the 50 eligible drug–DI pairs with the highest crude ROR values after requiring at least three FAERS DI-related reports.

For the frequency-ranked top 50 non-treatment PS drugs, we calculated crude ROR, covariate-adjusted ROR, EBGM, and IC. Adjusted RORs were estimated using multivariable logistic regression with PT-defined DI status as the dependent variable and drug exposure as the independent variable of interest (Bae et al., 2021; Hoffman et al., 2012; Ji et al., 2022). FAERS models adjusted for age group, sex, reporting period, country or region group, reporter type, presence of an outcome code, number of PS drug records, total number of reported drug records, and number of adverse-event terms. JADER models adjusted for age group, sex, reporting period, reporter type, report type, number of PS drug records, total number of reported drug records, number of adverse-event terms, and number of reported indications, where available.

EBGM was estimated using a single-gamma Gamma–Poisson empirical Bayes shrinkage model. EB05 and EB95 were defined as the 5th and 95th percentiles of the posterior distribution. IC analysis was performed using a 0.5 shrinkage continuity correction, with IC025 and IC975 estimated on the IC scale. Positive signals were defined as follows: crude ROR and adjusted ROR, at least three reports with lower 95% CI greater than 1; EBGM, at least three reports with EB05 ≥ 2; and IC, at least three reports with IC025 greater than 0. A proportional reporting ratio (PRR) analysis was retained as a supplementary sensitivity analysis.

### 2.8 Sensitivity analyses and cross-database assessment

Sensitivity analyses were conducted to examine the robustness of the main signal pattern. These included PS + SS drug-role sensitivity analysis, ROR-ranked top-50 prioritization, PRR-based sensitivity analysis, exclusion of DI diagnostic or treatment drugs during initial preprocessing, normalized annual reporting proportion analysis, subtype-oriented MedDRA coding assessment, and clinical-context sensitivity analysis using available indication, history, and co-reported adverse-event fields.

To further examine potential confounding by indication, perioperative or critical-care context, and non-drug etiologies, we performed an additional clinical-context sensitivity analysis. For FAERS, the newly harmonized full-period INDI table and co-reported adverse-event terms in the REAC table were screened using predefined keyword categories. For JADER, indication terms, drug-table indication terms, and co-reported adverse-event terms were screened using the same categories, including DI or AVP-deficiency indications, pituitary or hypothalamic disease, cranial injury or neurosurgical/intracranial disease, and perioperative or critical-care context. Reports meeting these criteria were removed from the PT-defined DI case set, and crude ROR, adjusted ROR, EBGM, and IC analyses were recalculated for the same FAERS frequency-ranked top 50 non-treatment primary suspect drugs. This analysis was conservative and exploratory because the available structured fields cannot fully distinguish baseline disease, clinical setting, and drug-induced events.

For cross-database assessment, the same FAERS frequency-ranked top 50 non-treatment PS drugs were evaluated in JADER. JADER reports were identified using the same PT-defined DI endpoint and the same treatment-drug exclusion strategy. Drug-name mapping between FAERS and JADER was manually reviewed using standardized active ingredient fields where available. Drugs with no JADER DI-related reports were retained in tabular summaries as having no JADER DI-report support and were not interpreted as replicated signals.

Cross-database signal reproducibility was assessed using crude ROR, adjusted ROR, EBGM, and IC calculated within JADER. Robust cross-database support was defined as positivity in at least three of the four JADER methods. Log-scale correlation analyses included only drugs with positive finite estimates in both databases for the metric being plotted. Drugs with zero or non-estimable JADER values were excluded from log-scale scatter plots and reported separately.

All analyses were interpreted as pharmacovigilance signal-detection analyses. Covariate adjustment and shrinkage-based metrics were used to improve signal stability and reduce the influence of observable covariate imbalance or sparse cells, but they cannot establish causality, estimate incidence, or fully address unmeasured confounding, reporting bias, or the absence of exposure denominators.

### 2.9 PubMed-based systematic case-level review and proportional synthesis

**FIGURE 2:**
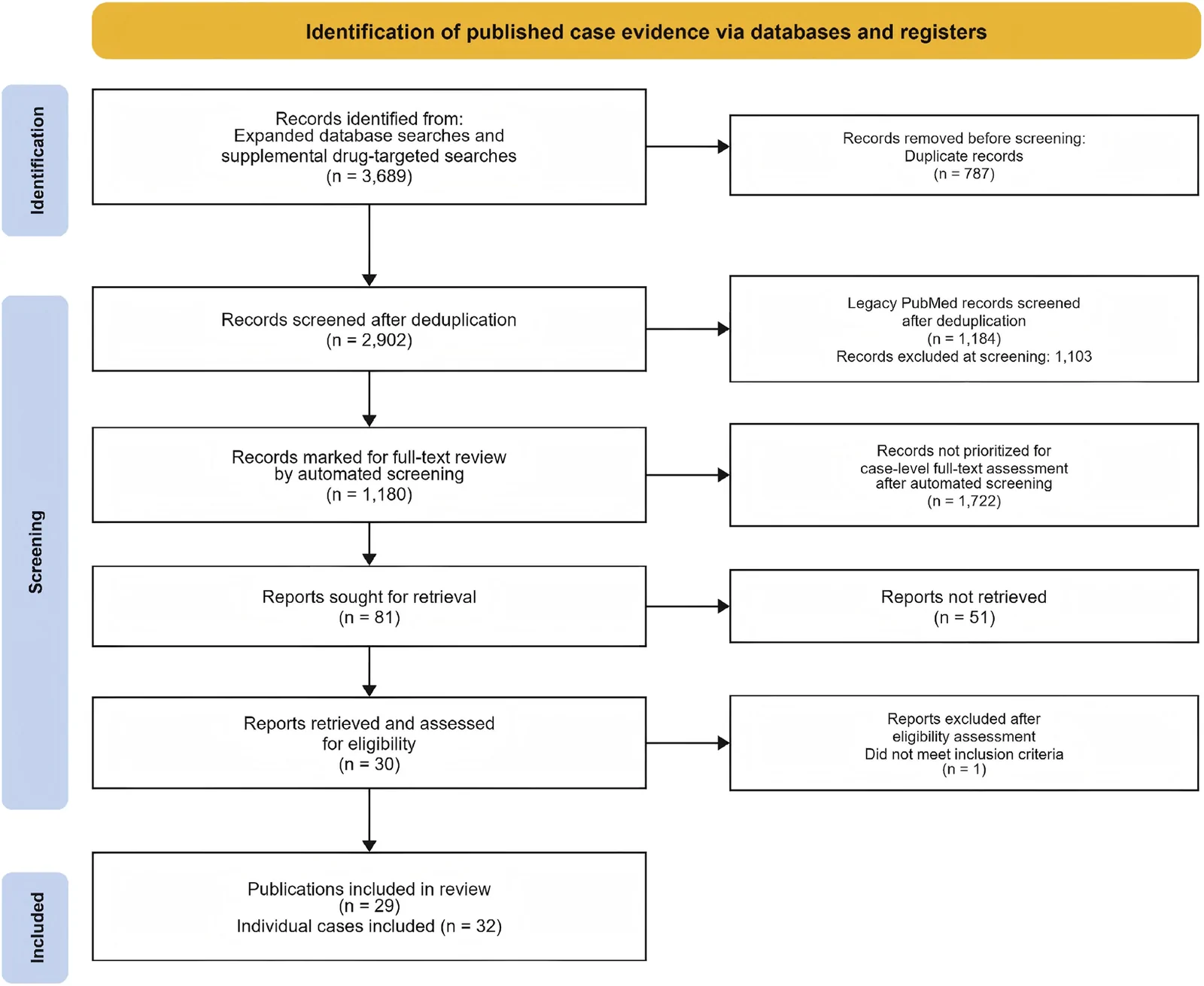
PRISMA flow diagram for published case-level evidence. Flow diagram showing the identification, screening, retrieval, eligibility assessment, and inclusion of published case-level evidence on suspected drug-associated diabetes insipidus (DI) or DI-like polyuria. Expanded database searches and supplemental drug-targeted searches identified 3,689 records, of which 787 duplicates were removed. After deduplication, 2,902 records were screened, and 1,180 records were marked for full-text review by automated screening. Eighty-one reports were sought for retrieval, 30 were retrieved and assessed for eligibility, and 29 publications comprising 32 individual cases were included in the systematic case-level review. The published-case synthesis was used to summarize extractable clinical features and diagnostic evidence, rather than to estimate incidence, comparative risk, or causality.

To provide clinical context for the pharmacovigilance findings, we conducted a systematic case-level review of published reports of suspected drug-associated DI or DI-like polyuria. PubMed/MEDLINE, CrossRef, and OpenAlex were searched from database inception to 19 March 2026 using terms related to diabetes insipidus, central diabetes insipidus, nephrogenic diabetes insipidus, AVP deficiency or resistance, drug-induced adverse reactions, and case reports. Records were deduplicated by DOI, PMID, and normalized title (Figure 2).

Eligible publications were case reports or case series that described suspected drug-associated DI or DI-like polyuria and provided extractable individual patient-level information. Reviews without original cases, non-human studies, pharmacovigilance database-only studies, conference abstracts without sufficient clinical detail, and reports in which DI was only a pre-existing condition or treatment indication were excluded. Extracted variables included suspected drug, DI subtype, time to onset, urine output, serum sodium, urine osmolality or specific gravity, serum osmolality, AVP or copeptin findings, desmopressin or vasopressin response, dechallenge, rechallenge, clinical outcome, and causality assessment.

The screening, extraction, and quality-appraisal workflow followed standard systematic-review procedures, including duplicate screening and extraction, adjudication of discrepancies, and structured critical appraisal using the JBI framework for case reports and case series. Because the eligible evidence consisted mainly of case reports and case series without exposed population denominators, comparator groups, or person-time, a conventional comparative risk meta-analysis was not feasible and was not attempted. We therefore performed a proportional synthesis of extractable clinical features among published cases. For endpoints with sufficient variation, pooled proportions were estimated using a random-effects logit model. When all included studies for an endpoint had zero events or all events, an aggregate proportion with Wilson 95% CI was reported instead because random-effects pooling was not informative. These estimates describe the distribution of features among published cases and were not interpreted as incidence, absolute risk, or causal effect estimates.

## 3 Results

### 3.1 Reporting trend and demographic characteristics of DI-related reports

From 2004Q1 to 2025Q4, 23,866,920 original individual case safety reports (ICSRs) were retrieved from FAERS. After case-level deduplication based on CASEID, FDA_DT, and PRIMARYID, 20,550,043 unique reports were retained for the overall database description. For normalized annual trend calculations, 20,149,410 deduplicated reports with an analyzable reporting year contributed to the annual denominator. Among the deduplicated reports, 3,444 contained the target MedDRA Preferred Terms for DI, including “Diabetes insipidus” and “Nephrogenic diabetes insipidus.” After restriction to reports with at least one primary suspect drug, 3,398 DI-related reports were available for primary-suspect drug descriptive analyses. After excluding drugs used for DI diagnosis or treatment or AVP-deficiency states during primary-suspect drug preprocessing, 3,325 PT-defined DI-related FAERS reports with at least one remaining primary suspect drug entered the main non-treatment-drug signal analysis.

The MedDRA term audit showed that central DI could not be isolated as a distinct PT-level endpoint in either FAERS or JADER. Central DI-related lower-level terms were mapped to the broader PT “Diabetes insipidus,” whereas “Nephrogenic diabetes insipidus” was available as a distinct PT. In FAERS, among 3,444 PT-defined DI-related reports, 2,318 reports (67.31%) contained only the non-subtype-specific PT “Diabetes insipidus,” 1,109 reports (32.20%) contained only “Nephrogenic diabetes insipidus,” and 17 reports (0.49%) contained both PTs. The database-level PT subtype distribution further supported this coding pattern (Supplementary Figures S1, S2).

**FIGURE 3:**
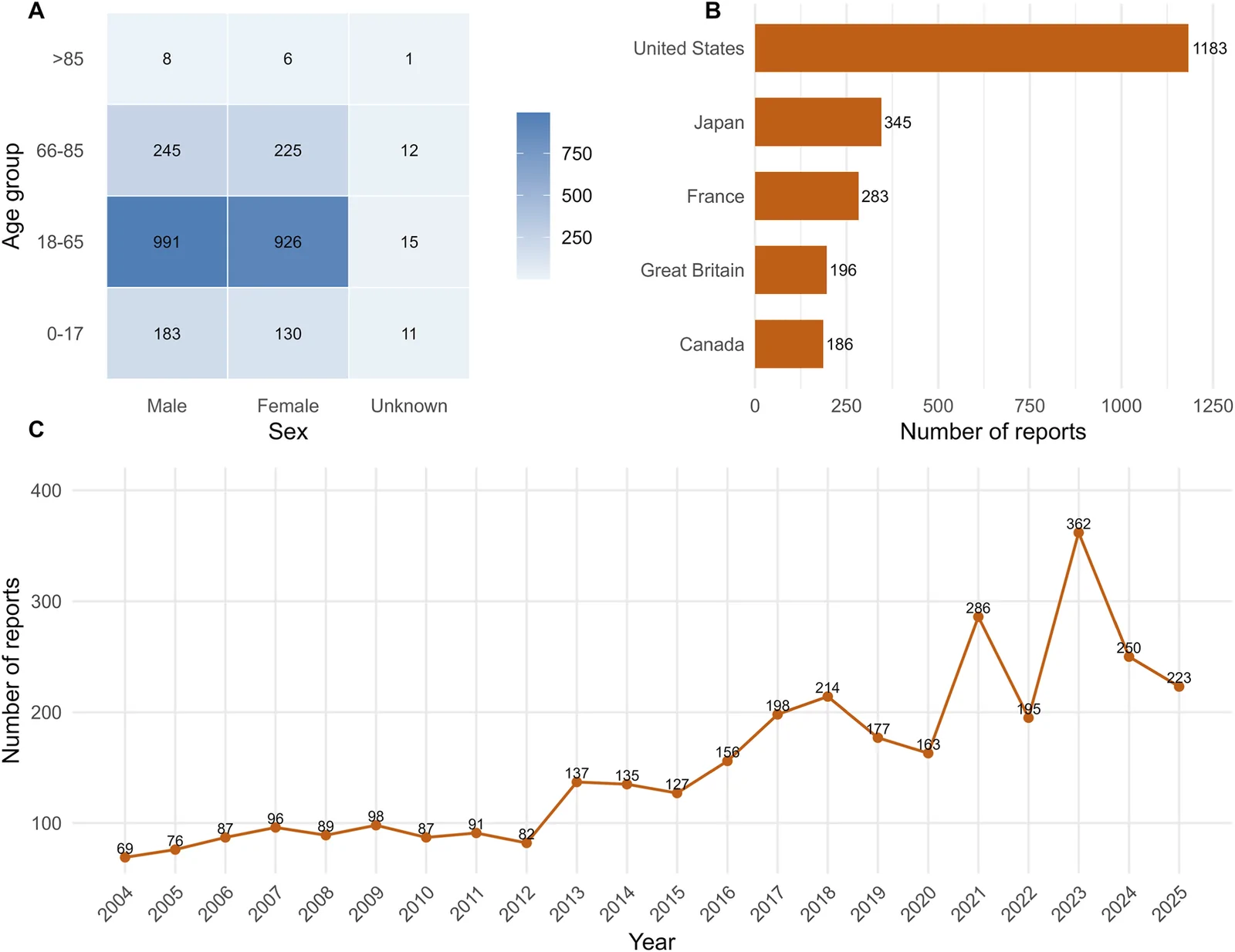
Demographic, geographic, and temporal distribution of diabetes insipidus-related reports in FAERS from 2004 to 2025. **(A)** Heat map showing the distribution of diabetes insipidus (DI)-related individual case safety reports by age group and sex. Numbers within each cell indicate the number of reports in the corresponding age–sex subgroup. **(B)** Distribution of DI-related reports across the five most frequently reported countries or regions. Numbers beside the bars indicate report counts. **(C)** Annual number of DI-related reports submitted to FAERS between 2004 and 2025.

The absolute annual number of PT-defined DI reports increased from 69 in 2004 to 223 in 2025, with the highest count observed in 2023 (n = 362). However, when expressed per 100,000 deduplicated FAERS reports, the annual DI reporting proportion fluctuated rather than increasing monotonically. The normalized proportion was the highest in 2007 (38.60 per 100,000 reports) and was 13.79 per 100,000 reports in 2025 (Figure 3).

Among the 3,398 DI-related reports in the primary-suspect descriptive set, 2,753 reports had available age information. The 18–65-year age group accounted for the largest proportion (n = 1,932; 70.18%), followed by the 66–85-year group (n = 482; 17.51%). Reports involving patients aged 0–17 years accounted for 11.77% (n = 324), whereas those involving patients aged >85 years accounted for 0.54% (n = 15). Age was missing or unavailable in 645 reports.

For consistency with the age–sex demographic summary, sex was summarized within the same 2,753-report subset. Male patients accounted for 51.83% of reports (n = 1,427), female patients accounted for 46.75% (n = 1,287), and sex was unspecified in 1.42% (n = 39). Among reports with available reporter-country information (n = 3,186), the largest number originated from the United States (n = 1,183; 37.13%), followed by Japan (n = 345; 10.83%), France (n = 283; 8.88%), Great Britain (n = 196; 6.15%), and Canada (n = 186; 5.84%). Reporter country was missing or unavailable in 212 reports.

Regarding adverse-event outcomes recorded in the OUTC table, 3,367 reports had at least one outcome code. Other serious medical events (OT) constituted the most frequently reported category (n = 2,477; 73.57%), followed by hospitalization or prolonged hospitalization (HO; n = 1,753; 52.06%). Death (DE) was recorded in 309 reports (9.18%), and life-threatening outcomes (LT) were recorded in 306 reports (9.09%). Because multiple outcome codes can be recorded for a single report, outcome categories were not mutually exclusive. Outcome information was unavailable in 31 reports.

### 3.2 Drug distribution and therapeutic spectrum

**FIGURE 4:**
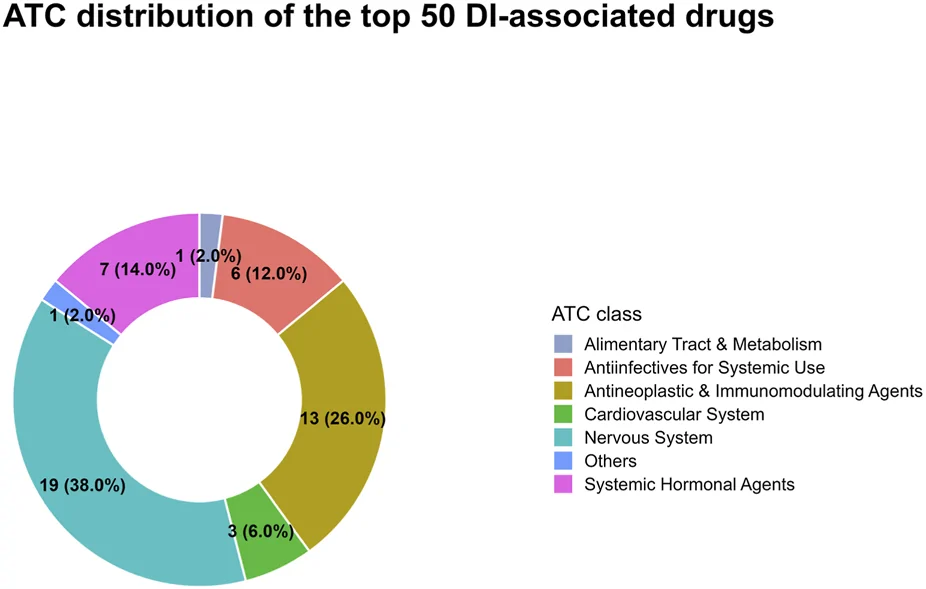
ATC distribution of the frequency-ranked top 50 FAERS non-treatment drugs associated with diabetes insipidus. The donut chart shows the distribution of the 50 non-treatment primary suspect drugs with the highest number of diabetes insipidus (DI)-related reports in FAERS after exclusion of drugs used for DI diagnosis or treatment. Drugs were grouped according to first-level Anatomical Therapeutic Chemical (ATC) classification. Labels indicate the number and percentage of drugs in each therapeutic category among the frequency-ranked top 50. Nervous system agents accounted for the largest proportion, followed by antineoplastic and immunomodulating agents, systemic hormonal agents, anti-infectives for systemic use, cardiovascular system drugs, and other categories.

Among the 50 remaining primary suspect drugs with the highest number of DI-related reports in FAERS after exclusion of DI diagnostic or treatment drugs, the distribution across first-level Anatomical Therapeutic Chemical (ATC) categories was uneven (Figure 4). Nervous system agents constituted the largest proportion, accounting for 38.0% of the top 50 drugs (n = 19), followed by antineoplastic and immunomodulating agents (n = 13; 26.0%) and systemic hormonal agents (n = 7; 14.0%). Anti-infectives for systemic use represented 12.0% (n = 6), cardiovascular system drugs accounted for 6.0% (n = 3), and alimentary tract and metabolism drugs and drugs classified as others each accounted for 2.0% (n = 1) (Figure 4).

At the individual-drug level, the nervous system category included lithium, dexmedetomidine, quetiapine, sevoflurane, valproic acid, olanzapine, clozapine, levetiracetam, aripiprazole, risperidone, ketamine, carbamazepine, lamotrigine, venlafaxine, haloperidol, phenytoin, propofol, diazepam, and fentanyl. The antineoplastic and immunomodulating category included temozolomide, methotrexate, pembrolizumab, carboplatin, ifosfamide, pemetrexed, nivolumab, tacrolimus, mycophenolic acid, ipilimumab, bendamustine, infliximab, and adalimumab. Systemic hormonal agents included hydrocortisone, somatropin, dexamethasone, prednisone, prednisolone, octreotide, and levothyroxine. Anti-infectives included doxycycline, tenofovir, amphotericin B, piperacillin, tazobactam, and emtricitabine, whereas cardiovascular drugs included tolvaptan, furosemide, and hydrochlorothiazide. Overall, after treatment-drug exclusion, the drug spectrum underlying DI-related reports remained concentrated mainly in nervous system agents, antineoplastic and immunomodulating therapies, and endocrine-related drugs.

### 3.3 Disproportionality analysis and frequency-ROR distribution in FAERS

All eligible non-treatment primary suspect drugs were screened for DI disproportionality before the top-50 summary was generated. In the overall FAERS PS-only disproportionality analysis after exclusion of DI diagnostic or treatment drugs, 112 drug–DI pairs met the crude ROR signal criterion of at least three DI reports and a lower 95% confidence interval greater than 1. A complete supplementary table lists these FAERS-positive pairs together with crude ROR, covariate-adjusted ROR, EBGM, IC, and JADER cross-database assessment results. The earlier crude-only audit table generated before the standardized four-method expansion identified 113 crude-positive pairs; reconciliation showed that the discrepancy was attributable to subsequent drug-name standardization and minor boundary changes in the crude ROR confidence interval for sparse pairs. Because some high-ROR combinations were supported by small report counts, the main figures focus on the 50 remaining drugs with the highest DI-related reporting frequencies to prioritize signals supported by greater report counts, rather than to imply that signal detection was limited to only 50 drugs (Supplementary Table S1).

**FIGURE 5:**
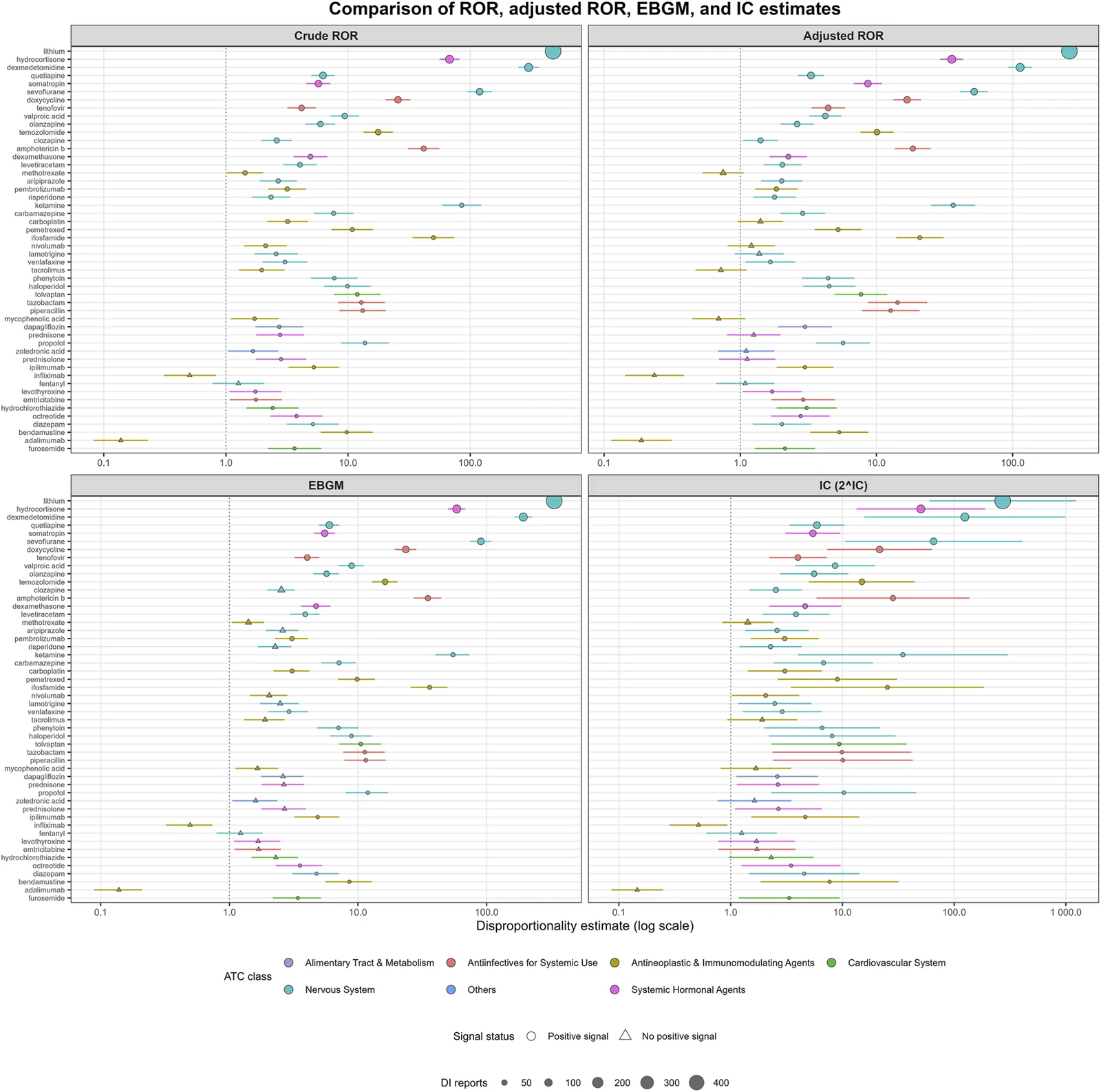
Multi-method disproportionality assessment of the frequency-ranked top 50 FAERS non-treatment primary suspect drugs associated with diabetes insipidus. The four-panel forest-bubble plot compares crude reporting odds ratio (ROR), covariate-adjusted ROR, empirical Bayes geometric mean (EBGM), and information component transformed to the reporting ratio scale [IC (2^IC^)] for the 50 non-treatment primary suspect drugs with the highest number of diabetes insipidus (DI)-related reports in FAERS. Drugs used for DI diagnosis or treatment were excluded before signal calculation and top-50 selection. The x-axis is displayed on a logarithmic scale, and the vertical dotted line indicates the null value of 1.0. Points indicate the corresponding disproportionality estimate, horizontal lines indicate 95% confidence intervals for ROR estimates or uncertainty intervals for shrinkage-based estimates, point size represents the number of FAERS DI-related reports, and point color represents first-level Anatomical Therapeutic Chemical (ATC) class. Point shape indicates whether the predefined signal criterion for each method was met.

Among these 50 frequency-ranked non-treatment drugs, 47 met the crude ROR criterion, 36 remained positive after multivariable adjustment, 31 met the EBGM criterion, and 39 met the IC criterion. Overall, 34 drugs were positive in at least three methods, and 30 were positive across all four methods (Figure 5; Supplementary Tables S2, S4). Ranked by crude ROR magnitude, lithium showed the strongest disproportionality signal (ROR, 483.85; 95% CI, 437.94–534.58), followed by dexmedetomidine (ROR, 294.60; 95% CI, 242.98–357.19), sevoflurane (ROR, 118.17; 95% CI, 93.94–148.67), hydrocortisone (ROR, 65.46; 95% CI, 54.21–79.04), and ifosfamide (ROR, 49.38; 95% CI, 33.26–73.30). The strongest adjusted and shrinkage-supported signals included lithium, dexmedetomidine, sevoflurane, hydrocortisone, ifosfamide, amphotericin B, doxycycline, and temozolomide.

**FIGURE 6:**
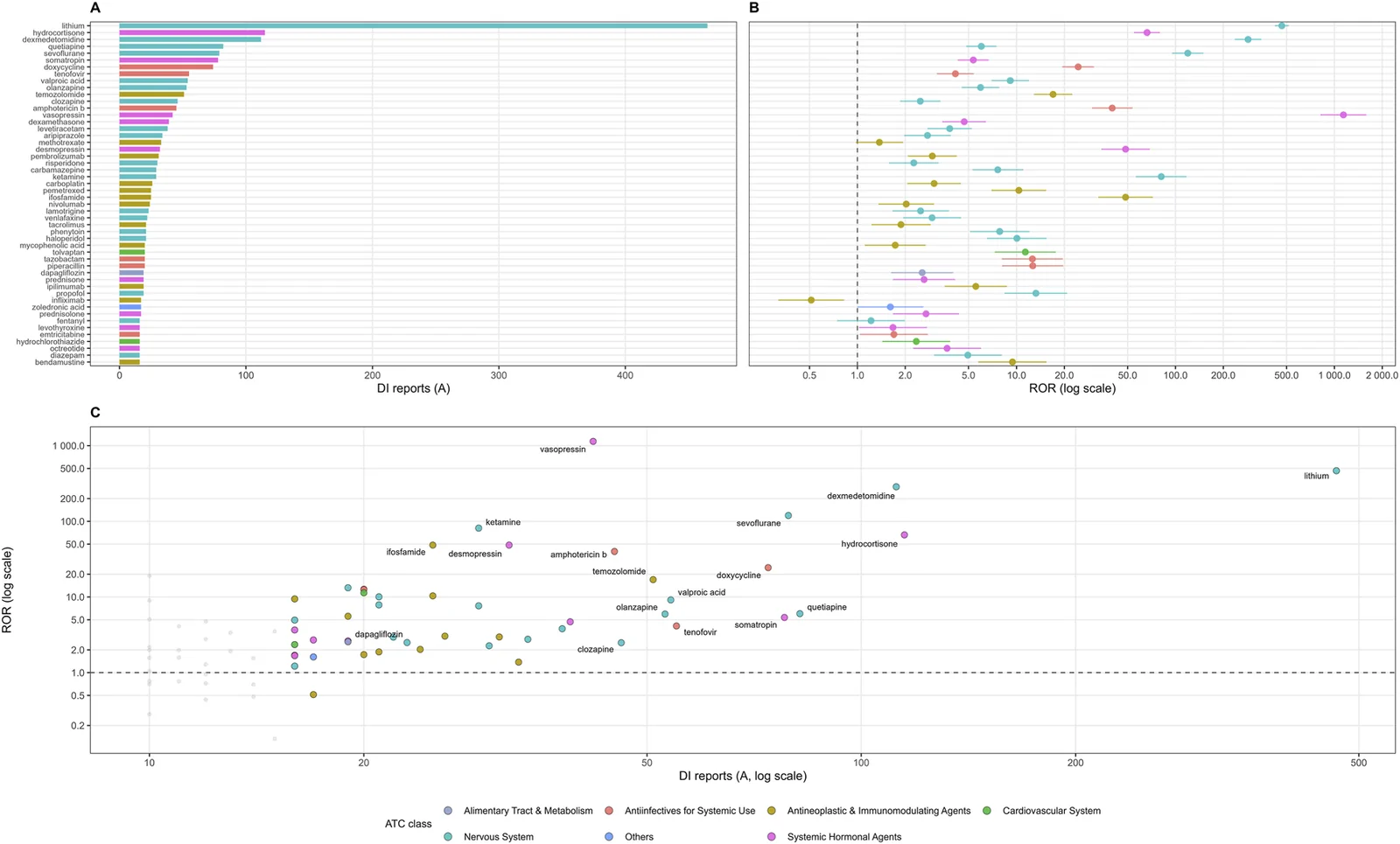
FAERS top 50 non-treatment drugs by diabetes insipidus-related report count, disproportionality estimates, and frequency-based selection context. **(A)** Number of DI-related reports for the top 50 non-treatment primary suspect drugs in FAERS, ranked by report count and colored according to the first-level ATC class. **(B)** Crude RORs and corresponding 95% confidence intervals for the same 50 drugs, displayed on a logarithmic scale. The dashed vertical line indicates the null value of ROR = 1. **(C)** Contextual distribution of report count and disproportionality among eligible non-treatment drugs in FAERS. Grey points represent all eligible drugs, whereas colored points highlight the top 50 drugs using the same ATC classification as in **(A,B)**.

Adjustment and shrinkage-based analyses attenuated some crude signals, particularly those more vulnerable to sparse-cell instability, while preserving a subset of higher-priority signals that remained positive across multiple analytic approaches. To contextualize the frequency-ranked top-50 selection, we examined the distribution of DI-related report counts and disproportionality estimates among eligible non-treatment drugs. Lithium had the highest number of DI-related reports, followed by hydrocortisone, dexmedetomidine, quetiapine, somatropin, sevoflurane, doxycycline, tenofovir, valproic acid, temozolomide, and olanzapine. The report-count versus disproportionality distribution showed that the frequency-ranked top 50 captured drugs with relatively frequent DI reporting, while several also showed strong disproportionality (Figure 6). The joint distribution of DI-related report frequency and disproportionality magnitude is further shown in the volcano-style plot (Supplementary Figure S3).

### 3.4 Cross-database assessment using the JADER database

**FIGURE 7:**
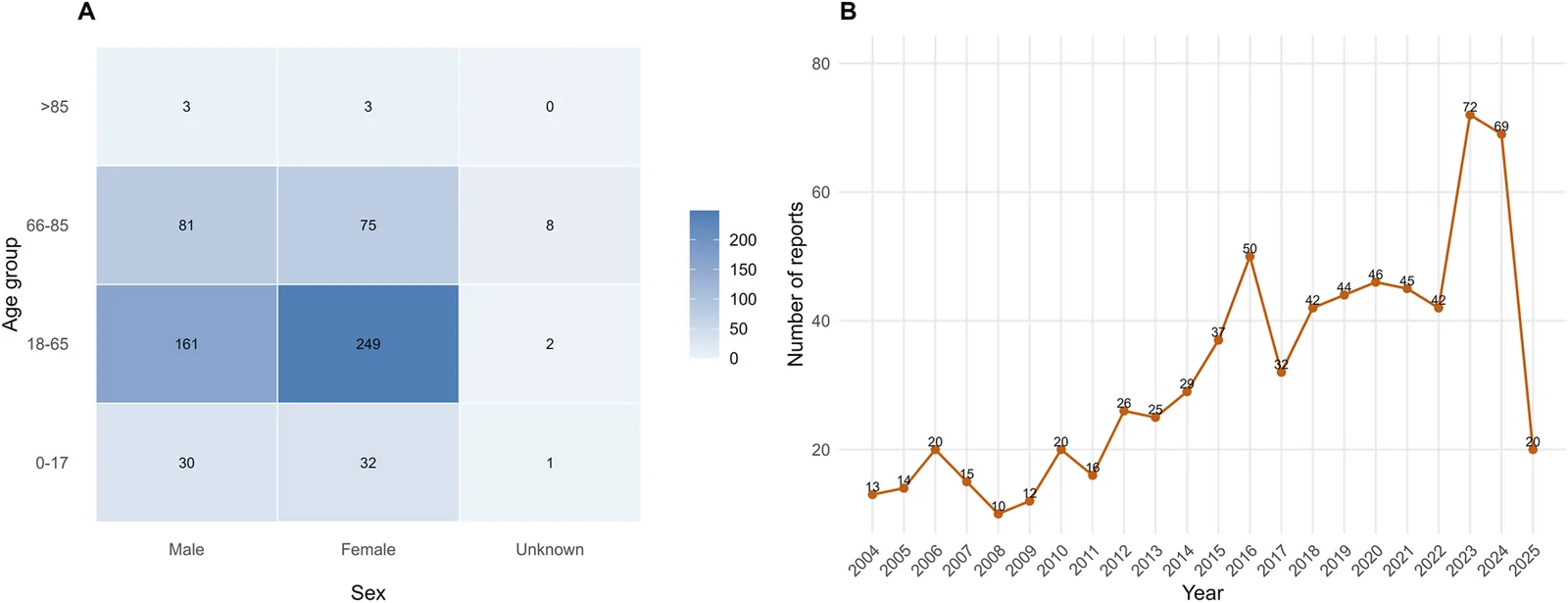
Demographic and temporal distribution of diabetes insipidus-related reports in the JADER database from 2004 to 2025. **(A)** Heat map showing the distribution of diabetes insipidus (DI)-related individual case safety reports by age group and sex in the Japanese Adverse Drug Event Report (JADER) database. Numbers within each cell indicate the number of reports in the corresponding age–sex subgroup. **(B)** Annual number of DI-related reports recorded in JADER between 2004 and 2025.

In JADER, 707 PT-defined DI-related reports were identified before restriction by drug role. After applying the primary-suspect drug restriction and excluding DI diagnostic or treatment drugs during drug preprocessing, 696 PT-defined DI reports entered the non-treatment-drug cross-database assessment. Descriptive age and sex patterns in JADER are shown in Figure 7; adult reports predominated, and the sex distribution showed a modest female predominance.

Cross-database assessment was performed in JADER for the 50 drugs included in the current FAERS frequency-ranked non-treatment top-50 analysis. Drug-name mapping was manually reviewed using the English standardized ingredient field in JADER. All 50 FAERS-ranked drugs were mapped to JADER drug names. Fourteen drugs had no corresponding JADER DI reports and were therefore described as having no JADER DI report support, rather than as externally reproduced signals.

**FIGURE 8:**
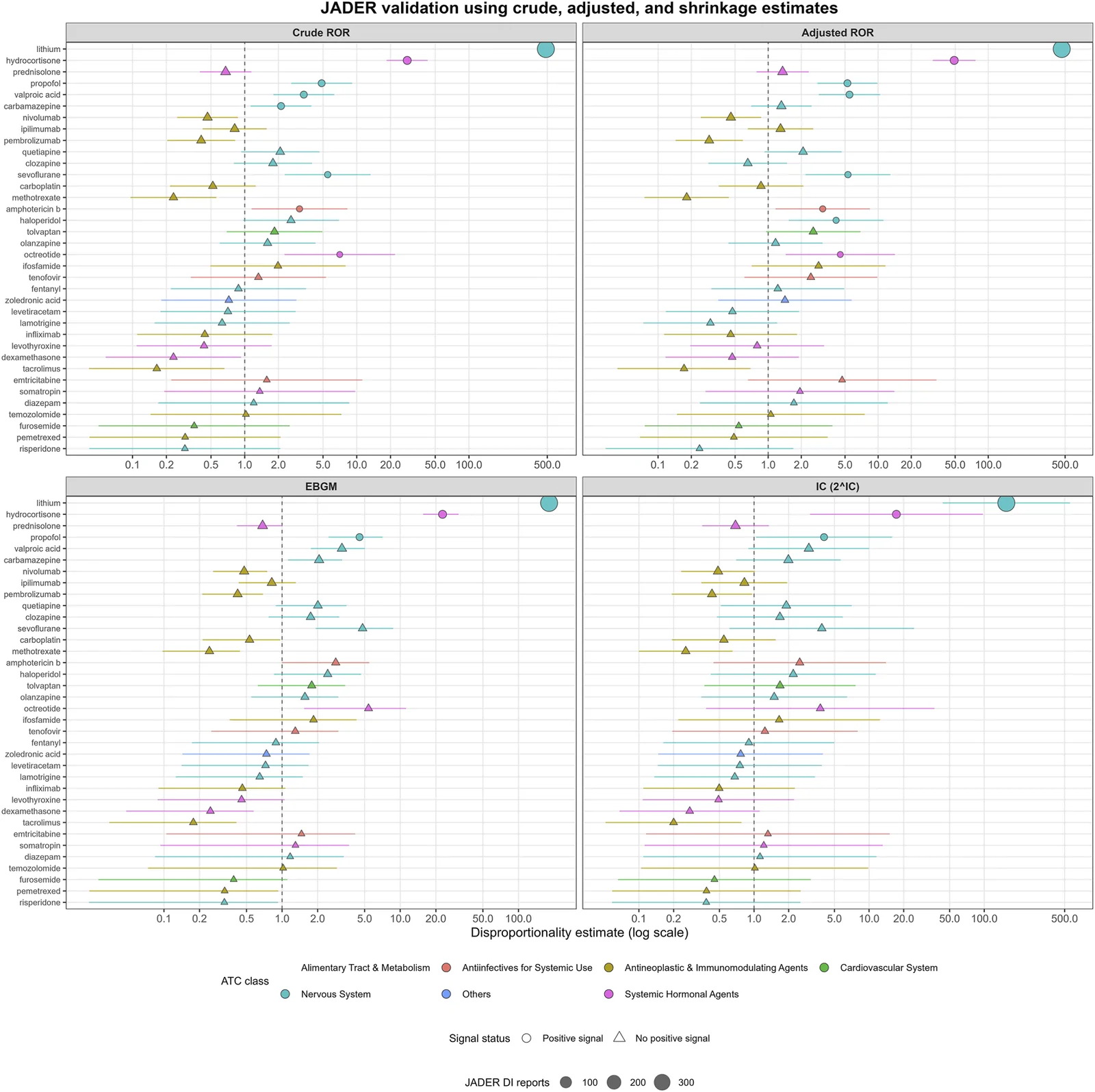
Cross-database assessment of FAERS-derived non-treatment diabetes insipidus signals in JADER. The four-panel forest-bubble plot shows crude ROR, covariate-adjusted ROR, EBGM, and IC transformed to the reporting ratio scale [IC (2^IC^)] calculated in JADER for drugs included in the FAERS frequency-ranked top-50 non-treatment primary suspect drug set. The x-axis is displayed on a logarithmic scale, and the vertical dashed line indicates the null value of 1.0. Points indicate the corresponding JADER disproportionality estimate, horizontal lines indicate 95% confidence intervals for ROR estimates or uncertainty intervals for shrinkage-based estimates, point size represents the number of JADER DI-related reports, and point color represents the first-level Anatomical Therapeutic Chemical (ATC) class. Point shape indicates whether the predefined signal criterion for each method was met. This analysis was used to assess cross-database signal reproducibility rather than to confirm causality.

Among the 50 evaluated drugs, eight showed positive crude ROR signals in JADER, eight remained positive after covariate adjustment, three met the EBGM criterion, and three met the IC criterion. Three drugs, lithium, hydrocortisone, and propofol, were positive across all four JADER methods and therefore met the prespecified robust cross-database signal-reproducibility criterion (Figure 8; Supplementary Tables S3, S4).

**FIGURE 9:**
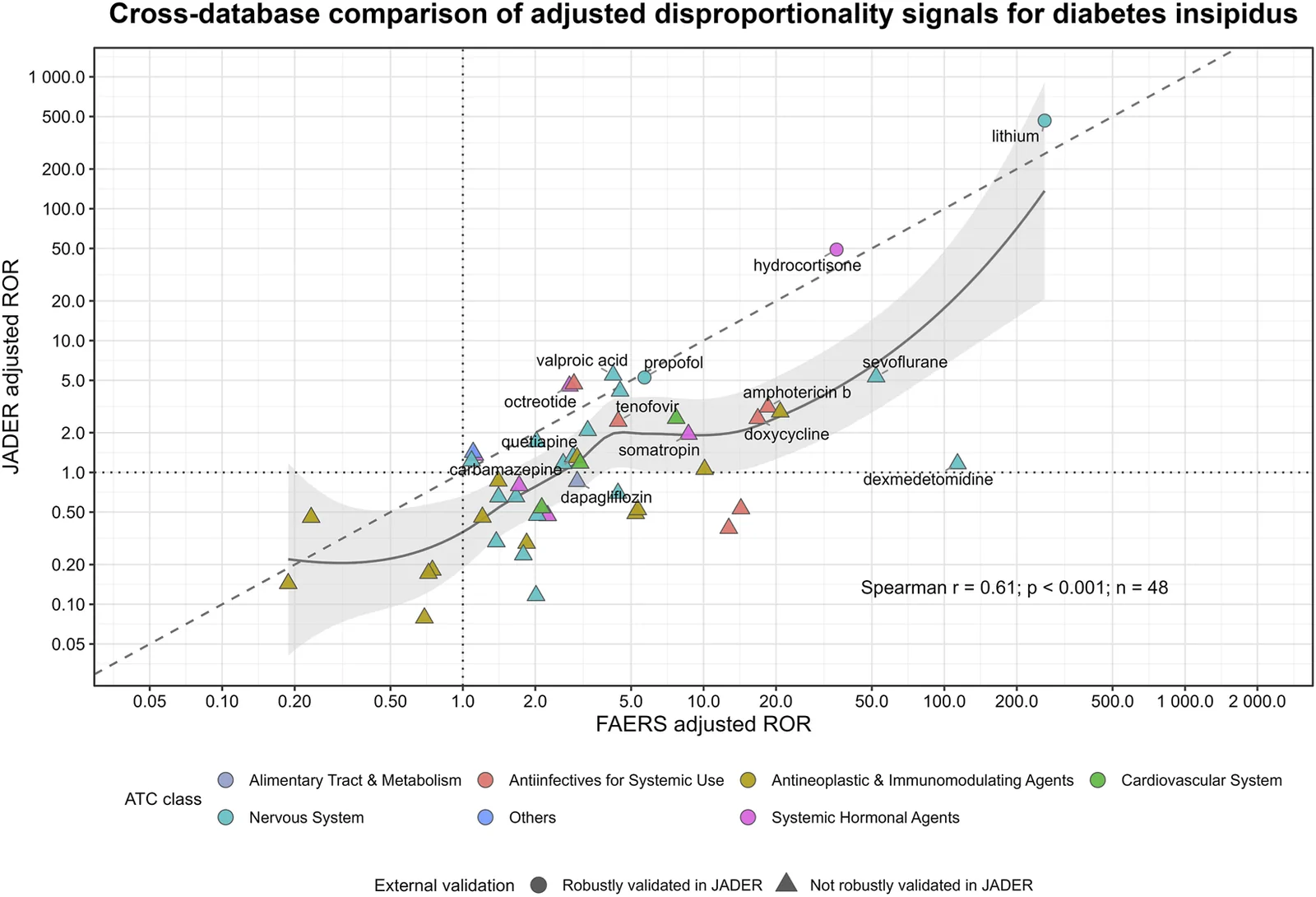
Cross-database comparison of diabetes insipidus-related adjusted disproportionality signals between FAERS and JADER. Scatter plot comparing adjusted reporting odds ratio (adjusted ROR) estimates between FAERS and JADER for 48 drugs with finite estimable adjusted ROR values in both databases among the 50 frequency-ranked non-treatment primary suspect drugs. Both axes are displayed on logarithmic scales. The x-axis shows FAERS adjusted ROR estimates, and the y-axis shows JADER adjusted ROR estimates. The dashed diagonal line indicates equal adjusted ROR estimates between the two databases, and the dotted reference lines indicate the null threshold of adjusted ROR = 1.0. Points are colored according to the first-level Anatomical Therapeutic Chemical (ATC) class. Point shapes indicate JADER cross-database support status; robust support was defined as positivity in at least three of the four JADER methods, namely, crude ROR, adjusted ROR, EBGM, and IC. The smoothed curve and shaded band show the overall trend and uncertainty. Spearman correlation was used to assess concordance in adjusted ROR magnitude across databases. Cross-database comparison reflects reproducibility of disproportionality patterns and does not imply causal confirmation.

Cross-database comparison was further performed using adjusted ROR estimates for drugs with finite log-scale adjusted estimates in both databases. Among the 50 mapped drugs, 48 were included in the log-scale scatter plot; ketamine and prednisone were not plotted because their JADER adjusted ROR estimates were zero or not estimable on the log scale. This scatter-plot denominator is distinct from the 14 drugs, with no observed JADER DI reports in the tabular assessment. FAERS and JADER adjusted ROR estimates showed a statistically significant moderate positive correlation (Spearman r = 0.61, p < 0.001; n = 48), suggesting partial cross-database concordance. However, the magnitude of adjusted ROR estimates varied substantially between databases, indicating that JADER should be interpreted as a cross-database assessment of signal reproducibility rather than quantitative confirmation of FAERS signal strength (Figure 9).

**FIGURE 10:**
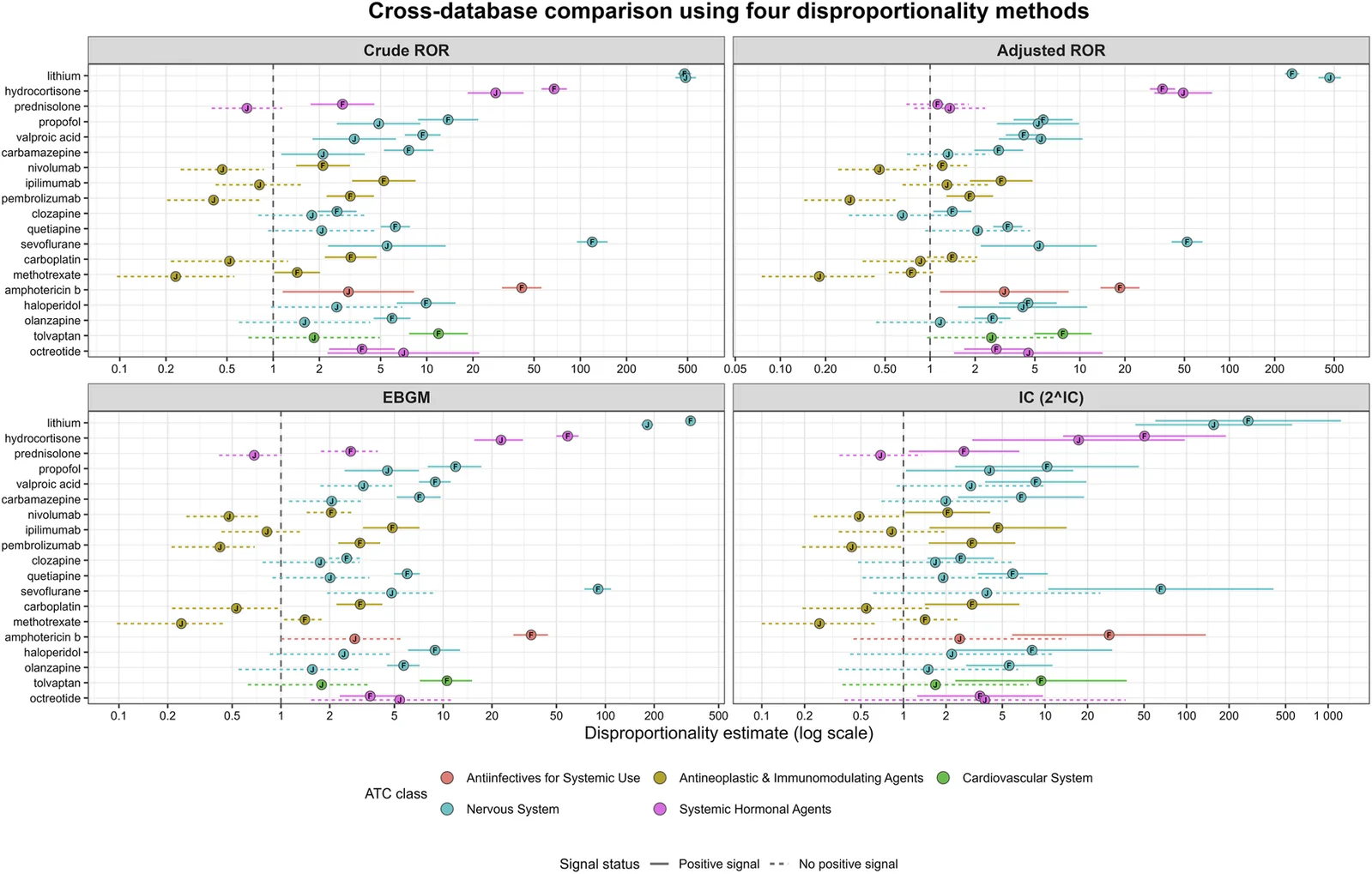
Cross-database comparison of diabetes insipidus-related disproportionality estimates using four analytic methods. Forest plot comparing FAERS and JADER disproportionality estimates for FAERS frequency-ranked top 50 non-treatment primary suspect drugs with at least three diabetes insipidus (DI)-related reports in JADER. The four panels show crude ROR, covariate-adjusted ROR, EBGM, and IC transformed to the reporting ratio scale [IC (2^IC)^]. The x-axis is displayed on a logarithmic scale, and the dashed vertical line indicates the null value of 1.0. Points represent database-specific estimates, with within-point labels indicating the database source: F for FAERS and J for JADER. Horizontal lines represent 95% confidence intervals for ROR-based estimates or uncertainty intervals for EBGM and IC-based estimates. Solid interval lines indicate positive signals, whereas dashed interval lines indicate non-positive signals according to the corresponding method-specific criterion. Colors indicate the first-level Anatomical Therapeutic Chemical (ATC) class. This comparison summarizes cross-database consistency across crude, adjusted, and shrinkage-based disproportionality methods and should not be interpreted as causal confirmation.

A four-method comparative forest plot was generated for FAERS frequency-ranked top 50 drugs that had at least three DI-related reports in JADER. This plot compared crude ROR, adjusted ROR, EBGM, and IC estimates across FAERS and JADER. Lithium showed strong disproportionality in both databases across all four methods, consistent with its established association with nephrogenic DI. Hydrocortisone and propofol also remained positive across all four JADER methods. Several other drugs, including sevoflurane, valproic acid, amphotericin B, carbamazepine, haloperidol, and octreotide, showed partial support in one or two JADER methods but did not meet the robust multi-method reproducibility criterion (Figure 10).

Overall, only a small subset of FAERS frequency-ranked top 50 non-treatment drugs met the strict JADER cross-database robustness criterion. This result does not invalidate the FAERS screening analysis, but it indicates that crude ROR thresholds are permissive and that adjusted and shrinkage-based analyses are useful for signal prioritization. Differences may also reflect cross-national variation in prescribing practices, time since market approval, population characteristics, drug availability, coding practices, and spontaneous reporting behavior.

### 3.5 Sensitivity and signal-stability analyses

As an alternative signal-prioritization strategy, we performed an ROR-ranked top-50 sensitivity analysis after excluding DI diagnostic or treatment drugs and requiring at least three FAERS DI-related primary-suspect reports. Among the FAERS ROR-ranked top 50 drugs, 50 met the crude ROR criterion, 45 met the adjusted ROR criterion, 41 met the EBGM criterion, and 25 met the IC criterion. Overall, 40 drugs were positive in at least three methods, and 25 were positive across all four methods. In the JADER cross-database assessment of the same ROR-ranked set, 12 drugs met the crude ROR criterion, 12 met the adjusted ROR criterion, 6 met the EBGM criterion, and 5 met the IC criterion; six drugs were positive in at least three methods, and five were positive across all four methods. These findings show that crude ROR-based ranking can identify high-disproportionality pairs, but adjusted and shrinkage-based analyses are necessary to prioritize signals with greater stability (Supplementary Figures S4, S5).

The PS + SS sensitivity analysis broadened suspected-drug capture by including both primary and secondary suspect drugs after the same treatment-drug exclusion. In this analysis, 3,386 FAERS DI-related reports had at least one remaining primary or secondary suspect drug. At the drug level, 210 drugs met the crude ROR criterion, 132 met the adjusted ROR criterion, 138 met the EBGM criterion, and 115 met the IC criterion. The overlap between PS-only and PS + SS frequency-ranked top-50 drug lists indicated that the main frequency-ranked signal spectrum was broadly stable but not identical when secondary suspect drugs were included (Supplementary Figures S6, S7).

For the frequency-ranked non-treatment top 50 drugs, the volcano-style visualization and PRR analysis were broadly consistent with the primary ROR findings. The volcano plot showed that most selected drugs combined relatively frequent DI reporting with elevated disproportionality, and the PRR sensitivity analysis supported the robustness of most ROR-positive signals (Supplementary Figures S3, S8).

In the clinical-context sensitivity analysis using available indication and co-reported adverse-event fields, 699 of 3,371 FAERS PT-defined DI reports in the PS-only analytic set were removed from the DI case set after being identified for possible DI/AVP-deficiency indication, pituitary/hypothalamic disease, neurosurgical/intracranial disease, or perioperative/critical-care context, leaving 2,672 FAERS DI reports for sensitivity analysis (Supplementary Figure S9). Overall, 720 FAERS PT-defined DI reports contained at least one clinical-context flag before restriction to the PS-only analytic set. In JADER, 95 of 696 DI reports were removed from the analytic set, leaving 601 reports; 100 JADER PT-defined DI reports contained at least one clinical-context flag. Among the same FAERS frequency-ranked top 50 non-treatment primary suspect drugs, 31 FAERS-assessed drugs and 3 JADER-assessed drugs remained positive in at least three of the four methods after clinical-context exclusion. The analysis should be interpreted as a conservative robustness check, rather than a definitive method, for eliminating confounding by indication or clinical context (Supplementary Figures S10, S11).

### 3.6 Systematic case-level review and proportional synthesis of published reports

The updated search and deduplication workflow identified 3,689 database records, of which 787 were removed as duplicates and 2,902 records remained after deduplication. Because many records represented reviews, pharmacovigilance analyses, or reports without extractable individual cases, the full-text case-level assessment focused on reports likely to contain original patient-level evidence. In the curated full-text assessment set, 81 reports were sought for retrieval, 30 were retrieved and assessed, and 29 publications comprising 32 individual case-level records were included (Figure 2).

The published case evidence was summarized as a systematic case-level synthesis rather than as a comparative risk meta-analysis. The included publications consisted mainly of case reports and small case series, and they did not provide exposed denominators, comparator populations, or person-time required for estimating pooled relative or absolute risk. Therefore, the PubMed evidence was used to describe clinical phenotype, diagnostic support, dechallenge or rechallenge information, outcomes, and causality assessment among published cases.

**FIGURE 11:**
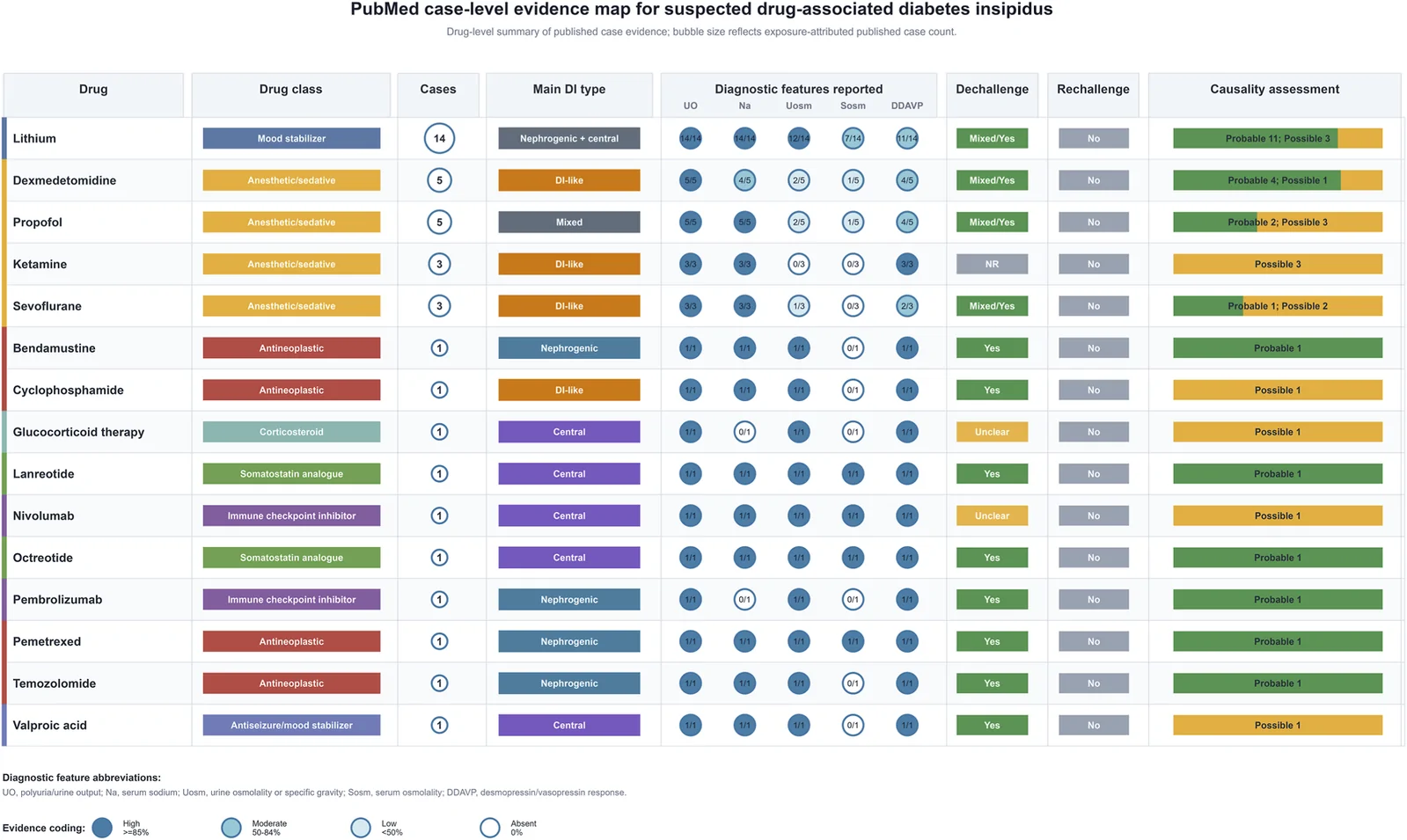
PubMed case-level evidence map for suspected drug-associated diabetes insipidus. Drug-level summary of published case evidence for suspected drug-associated diabetes insipidus (DI) or DI-like polyuria. Rows represent suspected drugs, and columns summarize drug class, exposure-attributed published case count, main reported DI phenotype, diagnostic features, dechallenge, rechallenge, and causality assessment. Bubble size in the “Cases” column reflects the number of exposure-attributed published cases for each drug. Diagnostic features are shown as the number of cases reporting each feature divided by the total number of published cases for that drug; filled circles indicate more frequently reported features, and open circles indicate absent or less frequently reported features. Abbreviations: UO, polyuria or urine output; Na, serum sodium; Uosm, urine osmolality or specific gravity; Sosm, serum osmolality; DDAVP, desmopressin or vasopressin response. The figure summarizes published case-level clinical evidence and should not be interpreted as estimating incidence, comparative risk, or causality.

The drug-level evidence map showed that lithium contributed the largest number of published cases, followed by anesthetic or sedative agents, including dexmedetomidine, propofol, ketamine, and sevoflurane (Figure 11). Among cases with extractable subtype information, nephrogenic DI, central DI, and DI-like or unclear phenotypes were all represented, supporting clinical heterogeneity rather than a single mechanism. Diagnostic documentation varied across reports, but urine output, serum sodium, urine osmolality or specific gravity, and desmopressin or vasopressin response were frequently reported for several drugs. Positive dechallenge was described in a subset of reports, whereas no positive rechallenge was documented. The proportional synthesis of published-case features is provided in the Supplementary Material and should be interpreted only as the distribution of features among published cases, not as incidence or risk.

## 4 Discussion

This study refines the pharmacovigilance assessment of drug-associated DI by focusing on non-treatment primary suspect drugs and evaluating signal stability across several analytic layers. After removal of drugs used for DI diagnosis or treatment during initial preprocessing, DI-related reports in FAERS remained concentrated among nervous system agents, antineoplastic and immunomodulating therapies, and systemic hormonal agents. Several signals persisted across crude, adjusted, and shrinkage-based methods, whereas only a small subset showed robust cross-database reproducibility in JADER. This pattern supports a tiered interpretation of signals, rather than a simple positive-versus-negative reading based on crude disproportionality alone.

The demographic and temporal findings should be interpreted in the context of spontaneous reporting. DI-related reports covered a broad age range and showed no marked sex imbalance, consistent with the heterogeneous clinical background of DI. Although raw annual FAERS counts increased over time, normalized reporting proportions fluctuated after accounting for overall FAERS reporting volume. Thus, temporal patterns are more appropriately interpreted as reporting patterns, rather than evidence of increasing disease occurrence or drug-related risk. Previous studies have indicated that DI can occur in children, adolescents, and adults (Christ-Crain et al., 2019) and that its age and sex distribution is influenced by the underlying etiological spectrum (Zhang et al., 2018). In the present analysis, most DI-related reports involved adults aged 18–65 years, whereas pediatric and very elderly patients accounted for smaller proportions. The relatively balanced sex distribution suggests that, at the reporting level, drug-associated DI signals were not strongly concentrated in either sex. The increase in reports over time may reflect multiple factors, including increased drug utilization, broader availability of relevant medications, heightened awareness of adverse event reporting, and cumulative expansion of the FAERS database.

The present findings partly align with, but also extend, the recent FAERS-based study by Lv et al. (2026). Both studies identified nervous system agents as a major therapeutic category and highlighted lithium and dexmedetomidine as prominent DI-related signals. The present analysis differs in several ways that affect interpretation. It extends FAERS follow-up to 2025Q4, uses a MedDRA term audit that includes both “Diabetes insipidus” and “Nephrogenic diabetes insipidus,” removes DI diagnostic or treatment drugs during preprocessing, restricts the main analysis to primary suspect drugs, adds adjusted ROR, EBGM, and IC estimates, and assesses signal reproducibility in JADER. These differences may explain why the exact number and ranking of signals differ from earlier FAERS-only screening studies.

The strongest evidence in this analysis supports lithium as an established drug-associated DI signal. Lithium is a recognized cause of nephrogenic DI, and it remained one of the strongest and most stable signals across crude, adjusted, and shrinkage-based analyses. Its consistency across FAERS, JADER, and published case-level evidence supports the internal validity of the signal-detection strategy. Clinically, lithium-associated DI should continue to be interpreted as a well-established renal concentrating defect rather than an emerging pharmacovigilance observation.

A second group includes biologically plausible signals with variable clinical support. Dexmedetomidine, sevoflurane, ketamine, and propofol may occur in perioperative or intensive-care settings where DI-like polyuria, altered AVP release, hemodynamic instability, neurosurgical disease, and critical illness can overlap (Jinnouchi et al., 2024). Ifosfamide and amphotericin B may be linked to renal tubular injury or impaired urinary concentrating capacity. Immune checkpoint inhibitors and selected antineoplastic or immunomodulating agents may be associated with pituitary, hypothalamic, or renal immune-related injury, although posterior pituitary involvement appears uncommon. These signals are clinically relevant for vigilance but should not be interpreted as confirmed causal relationships (Chen et al., 2024).

Because DI-related reports may occur in settings such as pituitary disease, neurosurgery, head trauma, perioperative care, and critical illness, we added a clinical-context sensitivity analysis using available structured indication and co-reported adverse-event terms. This analysis reduced the most obvious forms of clinical-context confounding but cannot fully separate drug causality from disease severity, intensive-care exposures, or incomplete reporting. Signals that persisted after this exclusion may be considered more stable for pharmacovigilance prioritization, whereas signals that attenuated should be interpreted with greater caution and may reflect the clinical setting in which the drug was used.

A third group consists of emerging or uncertain signals that were positive in FAERS but showed limited support after adjustment, shrinkage, cross-database assessment, or published case review. These findings are useful for pharmacovigilance prioritization because they identify drug–event combinations that merit closer monitoring. However, they should not be presented as established drug-induced DI risks. This distinction is especially important for drugs with low report counts, wide intervals, non-positive shrinkage metrics, or no JADER DI report support.

The JADER analysis provides a cross-database assessment of signal reproducibility rather than definitive confirmation. Only lithium, hydrocortisone, and propofol were positive across all four JADER methods. Several additional drugs showed partial support by crude or adjusted estimates but did not meet EBGM or IC criteria. This high non-reproducibility rate does not necessarily invalidate FAERS signals. Instead, it shows that crude ROR thresholds can be permissive and that cross-database, adjusted, and shrinkage-based analyses are useful for prioritizing signals with greater stability. Differences between FAERS and JADER may reflect drug availability, prescribing patterns, market penetration, coding practices, population structure, and national reporting behavior.

The MedDRA term audit also limits mechanistic interpretation. “Nephrogenic diabetes insipidus” was available as a distinct PT, whereas central DI-related lower-level terms were mapped to the broader PT “Diabetes insipidus.” Therefore, reports coded as “Diabetes insipidus” should be interpreted as non-subtype-specific DI, rather than definite central DI. Mechanistic explanations involving central AVP suppression or posterior pituitary dysfunction are, therefore, inferential and should be supported by case-level clinical evidence whenever possible.

The PubMed case-level synthesis adds clinical context but does not provide comparative risk evidence. Published cases supported a heterogeneous spectrum that included nephrogenic DI, central DI, and DI-like polyuria. Lithium had the strongest published case support, whereas anesthetic or sedative agents, immune checkpoint inhibitors, and selected antineoplastic or endocrine-related drugs were supported by smaller numbers of reports. Because case reports preferentially describe severe, unusual, or novel presentations, this evidence should be used to characterize phenotype and diagnostic plausibility rather than to estimate frequency or relative risk.

Clinically, these findings support an evidence-stratified approach to vigilance. For established associations such as lithium, clinicians should monitor urine output, serum sodium, renal function, hydration status, and symptoms of polyuria or polydipsia. In perioperative, intensive-care, oncology, and immunotherapy settings, new-onset hypotonic polyuria or unexplained hypernatremia should prompt review of recent drug exposure and competing etiologies. For emerging FAERS-only signals, the appropriate response is awareness and targeted follow-up, rather than causal attribution.

Further studies are needed to evaluate the higher-priority signals using designs that can address temporality and confounding more directly. Active-comparator new-user cohort studies could estimate relative and absolute risks for selected drugs. Self-controlled case series or case-crossover designs may help reduce time-invariant confounding when precise exposure and event dates are available. Electronic health record studies could add laboratory data, urine output, serum sodium, urine osmolality, renal function, drug dose, treatment duration, dechallenge, rechallenge, and competing diagnoses. Mechanistic studies are also needed to clarify whether specific signals reflect central AVP suppression, renal AVP resistance, aquaporin-2 dysregulation, tubular injury, or immune-mediated hypothalamic–pituitary damage (Wang Y. et al., 2025).

Overall, the revised analysis supports a cautious but clinically useful interpretation of drug-associated DI signals. The most informative signals were those that remained after treatment-drug exclusion and persisted across adjusted or shrinkage-based methods, especially when supported by biological plausibility, published cases, or cross-database reproducibility. The findings should, therefore, be considered hypothesis-generating pharmacovigilance evidence that can guide targeted clinical surveillance and future pharmacoepidemiologic research.

### 4.1 Limitations

Several limitations should be considered. First, FAERS and JADER are spontaneous reporting databases and are subject to under-reporting, duplicate reporting, incomplete information, and variable report quality (García-Abeijon et al., 2023; Costa et al., 2023; Hazell and Shakir, 2006). Because exposure denominators are unavailable, disproportionality analysis cannot establish causality, estimate incidence, or quantify absolute clinical risk (Bate and Evans, 2009; Faillie, 2019; Sa et al., 2013). Crude ROR, adjusted ROR, EBGM, and IC should, therefore, be interpreted as measures of disproportionate reporting or signal stability rather than true risk.

Second, reporting bias and confounding may have influenced the signal estimates. Although DI diagnostic or treatment drugs were excluded during initial preprocessing, residual confounding by indication, protopathic bias, comorbidity, concomitant medication use, Weber effects, notoriety bias, and stimulated reporting may still affect spontaneous reporting patterns (Kraus et al., 2023; Hoffman et al., 2014a; Hoffman et al., 2014b). Multivariable adjustment can reduce measured covariate imbalance, and EBGM or IC can reduce sparse-cell instability, but these methods cannot remove unmeasured confounding or prove causality.

Third, subtype-level interpretation was limited by PT-level coding. “Nephrogenic diabetes insipidus” was available as a distinct PT, whereas central DI-related lower-level terms were mapped to the broader PT “Diabetes insipidus” and were not separately available in public FAERS/JADER data. Thus, reports coded as “Diabetes insipidus” were interpreted as non-subtype-specific DI, rather than definite central DI. Key clinical details required for individual causality assessment, including temporality, dose, dechallenge, rechallenge, laboratory findings, renal function, and competing etiologies, were also inconsistently available.

Finally, the PubMed component had the limitations inherent to published case reports and case series. Although the screening, extraction, and appraisal workflow followed systematic-review procedures, the available evidence lacked exposed denominators, comparator groups, and person-time; therefore, a conventional comparative risk meta-analysis was not feasible. The proportional synthesis describes features among published cases only. Publication bias and selective reporting are likely because severe, unusual, diagnostically complete, or novel DI presentations are more likely to be published than expected or mild presentations. Therefore, the findings should be interpreted as hypothesis-generating pharmacovigilance signals, rather than confirmed causal drug–event relationships.

## 5 Conclusion

In this large-scale pharmacovigilance study, we characterized non-treatment drug-associated DI reporting signals in FAERS and assessed their consistency in JADER using cross-database assessment, adjusted disproportionality analysis, shrinkage-based methods, and structured published case-level evidence. After excluding drugs used for DI diagnosis or treatment during initial preprocessing, DI-related reports were concentrated mainly among nervous system agents, antineoplastic and immunomodulating agents, and systemic hormonal preparations. Several non-treatment drugs, including lithium, dexmedetomidine, sevoflurane, ketamine, hydrocortisone, ifosfamide, amphotericin B, and doxycycline, showed persistent disproportionality across crude, adjusted, and shrinkage-based analyses, whereas cross-database support in JADER was limited to a small subset. These findings may help prioritize potential drug-associated DI signals for clinical awareness and further investigation, but they should be interpreted as hypothesis-generating evidence requiring pharmacoepidemiologic, clinical, and mechanistic validation.

## Data Availability

The original contributions presented in the study are included in the article/Supplementary Material, further inquiries can be directed to the corresponding author.
